# Can Technology Abate the Experience of Social Isolation for Those Affected by Dementia?

**DOI:** 10.3389/fnagi.2021.779031

**Published:** 2022-02-22

**Authors:** Julie Faieta, Lily Faieta, Jean Leblond, François Routhier, Krista Best

**Affiliations:** ^1^Department of Rehabilitation, Université Laval, Quebec City, QC, Canada; ^2^Centre for Interdisciplinary Research in Rehabilitation and Social Integration, Centre Intégré Universitaire de Santé et de Services Sociaux de la Capitale-Nationale, Quebec City, QC, Canada; ^3^Pensacola Christian College, Pensacola, FL, United States

**Keywords:** dementia, caregiver, mobile device, applications, isolation

## Abstract

**Background:**

The widespread social isolation measures recently utilized to mitigate the spread of COVID-19 to older adults may have exuded unexpected consequences. Social isolation among older adults is a risk factor for poor health outcomes. Innovative solutions to balancing public safety and health maintenance for those with dementia and their caregivers are needed.

**Methods:**

A sample of *N* = 82 dementia caregivers participated in a web-based survey to investigate their perceptions on (1) changes in personal mental health due to isolation from their loved one, and (2) the perceived need for use of smart mobile device app use in these situations.

**Results:**

The majority of our sample (87%) reported experiencing negative mental health outcomes beyond those experienced in typical situations. Furthermore, over 70% of caregivers were concerned with the care their loved on received during social isolation. Finally, 67% reported perceived need to use SMD apps in these times of social isolation.

**Conclusion:**

Our findings provide preliminary insight into troubling consequences occurring when individuals with dementia are socially isolated from their caregivers. An inverse relationship between SMD app use and poor mental health points to the potential for communication technology to lessen the negative impacts of social isolation, when it becomes necessary to public safety.

## Introduction

The need for research specifically on social isolation measures between an individual with Alzheimer’s disease and related dementias (ADRD) and their informal caregiver is supported by an understanding of the way in which various forms of isolation, and the experience of loneliness that can result, negatively impact health outcomes. Research has shown that isolation and loneliness are positively associated with cardiovascular disease, diabetes, risky health behaviors, and poorer cognition (Shankar et al., 2013). Recent evidence has pointed to social isolation, in the more general sense, as one of 12 modifiable risk factors (lower education, hypertension, obesity, hearing loss, smoking, depression, low physical activity, social isolation, and diabetes) that contribute to approximately 35% of dementia cases (Livingston et al., 2017). Therefore, social distancing measures should be considered in light of both the health risks avoided *and* created by social isolation.

### Social Isolation and Dementia in the COVID-19 Pandemic

Social isolation has taken on a new meaning with the COVID-19 pandemic and the subsequent quarantine and social distancing public health measures implemented in both community and institutional settings across the globe. While initiated to lessen the spread of COVID-19, quarantine and social isolation procedures have in many cases led to the seclusion of vulnerable individuals, such as individuals with ADRD. Within this article, the term social isolation is primarily focusing on public health measures restricting hospital and extended care-facility visitation for extended periods, separating those with ADRD from their family members or informal caregivers. This drastic change in access to meaningful interpersonal relationships and social support cannot be overlooked. Furthermore, the social isolation measures designed to support safety, and reduce risk for the aging population, must be weighed against the potential negative impact that social isolation may have on quality of life.

The measurable impacts of social isolation in those with dementia should be further elucidated through improved understanding of the caregivers’ perspectives, as informal caregiver roles are commonly assumed by family members who advocate for the health and well-being of their loved one. The health of caregivers is measurably impacted (e.g., incidence of caregiver burden and negative mental health outcomes) by their role as a care provider (Brodaty et al., 2014; Park et al., 2015; Coffman et al., 2017; Liu et al., 2017). The unique context of strict social isolation with the onset of COVID-19 has the potential to impact both those with ADRD and caregivers in negatively. One might posit that separation from a care recipient could lessen burden of care and ongoing responsibilities for a caregiver. However, the negative outcomes of social isolation that result in separation of an informal caregiver (often a family member) from their loved one may instead produce worry and anxiety in the caregiver with regard to the well-being of their loved one. To improve the quality of life and care provision in conditions of social isolation, it is necessary to understand the perceptions of caregivers regarding the impact of social isolation from their loved ones, and the value of potential technology mediated solutions.

Technology use among older adults is increasingly prevalent (Anderson and Rainie, 2015; Pew Research Center, 2018, 2021). Therefore, it is an optimal point in time to increase research into the potential for technology mediated solutions for socially isolated older adults, to include those affected by dementia. van Boekel et al. (2019) carried out a systematic review of the available literature to investigate dementia stakeholder perspectives on technology use. Authors reported that technology use among those with dementia is facilitated by the potential for technology to prolong autonomy in the community setting (van Boekel et al., 2019). They also reported that technology use among dementia caregivers can help to alleviate stress and worry about their loved on with dementia (van Boekel et al., 2019). Both of these indicating the potential usefulness of technology mediated interventions to support both members of the ADRD dyad (caregiver and care recipient). Of interest, the authors note that the perspectives of community dwelling older adults with dementia aligned with the perspectives of healthy aging populations (van Boekel et al., 2019). Therefore, technology mediated communication to combat social isolation should be investigated in healthy aging, dementia, and caregiver populations.

The present study focuses on the impact of social isolation as perceived by ADRD caregivers. With increased use of smart mobile devices (SMD) across both age and socioeconomic demographics (Anderson and Rainie, 2015; Pew Research Center, 2018; Vogels, 2020), we will also investigate the use and perceptions of SMD apps as a possible solution to help compensate for physical separation through distance care and communication functions.

## Objective

The objectives of this study were to determine whether COVID-19 related social isolation between an individual with ADRD and a caregiver…

(1) Impacted caregiver mental health beyond what would be expected with caregiving in a typical context.
(2) Facilitated perceived need for use of smart mobile device (SMD) apps (e.g., video conference apps, messaging apps, browsers, etc.).

Our *a priori* hypotheses were (1) that social isolation related to the COVID-19 pandemic will be reported to increase poor mental health outcomes in caregivers beyond what is experienced in a typical context (e.g., non-COVID-19 isolation), and (2) that caregivers will report a perceived need for SMD app use during periods of social isolation. For the purpose of this study SMD refers to hardware that can house software (apps); the investigation is assessing use of apps, but inclusive of various types of SMDs such as smart phones or tablets pending participant preference.

## Materials and Methods

### Design

This study adheres to a cross-sectional design *via* a single time point web-based survey.

### Participants and Recruitment

Inclusion criteria included ≥18 years of age and the ability to read English or French (the survey was available in English and French) and providing informal care to an individual with ADRD. The definition of caregiver included spouses of individuals with dementia, adult children and other familial relations of individuals with dementia, and friends or other community care providers of individuals with dementia. In order to capture broad experiences of ADRD caregivers the COVID-19 pandemic, both caregivers who experienced social isolation from their loved ones and those who had not, or had not *yet* experienced social isolation from their loved one could respond. The prompt indicated that if they had not been socially isolated from their loved one, they could respond based on expected experiences. This approach allows recruitment of a broader sample of caregivers that is not contingent on current means, access, or inclination to place their loved one with ADRD in a care-facility–a choice that could be influenced by socioeconomic status, context, or cultural values.

An *a priori* power analysis was complete on G*Power 3.1.9.3 software considering *f*^2^ = 0.15, *p* = 0.05, and Power = 0.80, this yielded *N* = 68. Therefore, the target sample size was *N* = 82 participants to account for 20% attrition (attrition was anticipated to occur in this survey-based study when potential participants initiated the survey, but then discontinued due to inapplicability or lack of interest).

Email and social media were used for recruitment. Potential participants previously known to research personnel through existing networks and through rehabilitation and medical organization [i.e., AGE-WELL (a Canadian National Centre of Excellence on Technology and Aging Research), American Congress of Rehabilitation Medicine, and Quebec Health Research Network on Aging] list serves were contacted *via* email. Social media-based recruitment included posting to platforms such as LinkedIn, Twitter, and Facebook. Study methods were approved by the Centre Intégré Universitaire de Santé et de Services Sociaux de la Capitale-National Ethics Board (Ethics Approval No. CER 2020-1984).

### Procedures

The survey created for the purpose of this study included 10 items designed to investigate the relationship between the caregiver and care recipient, disease severity of the individual with dementia (i.e., mild, moderate, severe), the incidence of care recipient isolation due to COVID-19, need for app use (as perceived by caregiver), type of apps used by caregivers, and caregiver mental health outcomes (see Supplementary Appendix A). Multiple choice or Likert scale response options were provided to assess both person factors and experiential variables. The survey also allotted space for open comments for participants to share additional thoughts and experiences. The survey was managed using Lime Survey Software version 2.05+ (Limesurvey GmbH, 2020) and released online from May to September 2020. Participants completed the survey based on their experiences and perceptions as to how hospitalization of the person they care for during COVID-19 did *or* could have impacted them (as a caregiver) and the care recipient. All surveys were completed anonymously.

### Analyses

Survey results were analyzed using descriptive statistics (mean, standard deviation, frequency, proportion). A Principal Component Analysis (PCA) was used to evaluate the relationship of three independent variables (type of relationship to the individual with dementia, the disease severity of the individual with dementia, number of app types used) to the dependent variable of interest (number of negative mental health outcomes). The associations between these variables were explored using PCA for categorical data (SPSS, 26, proc CATPCA) to determine the most appropriate method of interpreting the data. While the classical PCA outputs loadings at the variable level, the categorical PCA outputs loadings for each category of each variable. Therefore, a graphical examination was then performed to determine whether the intervals between categories were equally spaced along the linear continuum associated to each variable. Further analyses were selected based on groupings of data (i.e., categories) that were closely located on the graph, and the slope of each continuum was considered to determine association (i.e., similar slopes indicative of a strong correlation and orthogonal slopes indicative of independence between variables) (Abdi and Williams, 2010). An ordinal regression model analysis (SPSS, 26, proc PLUM) was then completed to assess the influence of (1) relationship type (between caregiver and care recipient), (2) disease severity, and (3) extent of app use on number of mental health changes reported. Open-ended responses were summarized and documented, but no formal content analysis was conducted for the present report.

## Results

A total of 84 participants completed our survey (17 French surveys, 67 English). All participants were self-defined caregivers of an individual with ADRD. Of the 84 participants 54 (64.29%) were adult children providing care, 19 (22.62%) were spouses, 6 (7.14) were grandchildren, and 5 (5.95%) identified as other (to include a significant other, an art therapist, occupational therapist, and lifelong roommate) (see Table 1). Within our sample, 48 (57.14%) reported actively providing care to a person with ADRD, ranging from mild AD [9; (10.71%)]; moderate AD [29 (34.52%)]; and severe AD [31 (36.90%)] (see Supplementary Table 1).

**TABLE 1:** Perceived need for smart personal device.

	Frequency	Percent	Cumulative frequency	Cumulative percent
N/A	6	7.14	6	7.14
No	21	25.00	27	32.14
Yes	57	67.86	84	100.00

A total of 67 (79.76%) caregivers reported that their care recipient with ADRD was isolated due to institutionalization or hospitalization, and 60 (71.43%) caregivers were concerned about the care that medical or support personnel were able to provide to their care recipient. Sixty-eight (87.18%) caregivers reported experiencing negative health outcomes beyond what they normally experience during this period of isolation or fear of isolation associated with COVID-19.

The need for SMD app use during COVID-19 was indicated by 57 (67.86%) of respondents (see Table 2).

**TABLE 2:** Ordinal regression analysis.

*Estimates of parameters*								
		Estimation	Standard error	Forest	ddl	Sig.	95% confidence interval	
							Lower terminal	Upper terminal
Dependent	No neg. MH outcomes	–3.458	0.913	14.334	1	0.000	–5.248	–1.668
	1 neg. MH outcomes	–2.230	0.873	6.519	1	0.011	–3.942	–0.518
	2 neg. MH outcomes	–0.102	0.837	0.015	1	0.903	–1.742	1.539
Independent	Grandchild or Other relation Caregiver	–0.705	0.721	0.957	1	0.328	–2.117	0.707
	Adult Child Caregiver	–0.056	0.501	0.012	1	0.911	–1.037	0.925
	Spousal Caregiver	0^a^			0			
	Mild Dementia	–0.504	0.827	0.371	1	0.542	–2.126	1.118
	Moderate Dementia	–0.516	0.650	0.629	1	0.428	–1.791	0.759
	Severe Dementia	0.437	0.651	0.451	1	0.502	–0.839	1.713
	Other Dementia	0^a^			0			
	No apps	–2.133	0.618	11.923	1	0.001*	–3.344	–0.922
	1 type of app	–1.448	0.553	6.859	1	0.009*	–2.532	–0.364
	2–5 types of apps used	0^a^			0			

*Link function: Logit. ^a^This parameter is set to 0 because it is redundant.*

*Indicates significance.

The categorical PCA indicated that 67% of the variance of four variables represented two principal components (representing 36 and 31%). Graphical observation suggested that only “number of apps used” was related to mental health outcomes. The number of apps used was the only variable significantly associated to the number of negative health outcomes (Nagelkerke pseudo-*R*2 = 0.216, *p* = 0.009). Specifically, the likelihood of more negative health outcomes increased if no apps were used (95% CI, –3.344 to –0.922, *p* = 0.001), or only one app was used (95% CI, –2.532 to –0.364, *p* = 0.009), but did not increase significantly if 2 or more app types were used (see Table 2). It should be noted that model reported a Nagelkerke R squared of only 0.216, indicating that this model explains a small portion of the variance seen in mental health outcomes.

Caregivers who experienced additional anxiety during COVID-19 related isolation described their anxiety and fears about the potential that their loved was not being fed appropriately or that their loved one die could alone. Participants also discussed challenges with distance communication options, noting specific barriers such as inability to read facial expressions of their loved one with dementia, or the need to coordinate or attend to numerous calls (see Supplementary Appendix B for full statements).

## Discussion

Social isolation is a multi-dimensional experience that has been described according the following attributes: “loneliness, social support, social contact, number of confidants, social connectedness/social connectivity, social networks, and social well-being” (Chen and Schulz, 2016). Health concerns related to isolation among older adults are not novel to the COVID-19 context, as awareness and intervention continue to be of societal and research interests. Crewdson (2016) outlined the multifaceted outcomes related to loneliness among older adult populations to include psychiatric, behavioral, and physical outcomes, which may arguably holistically change one’s quality of life.

The sample population of caregivers was primarily comprised of adult children and spousal caregivers, both of whom are likely to have spent a fair portion of life with their loved one now living with ADRD. Most of those care recipients had moderate or severe ADRD and over 75% of those with ADRD were isolated due to institutionalization or hospitalization. It is critically important to highlight over 70% of caregivers reported that the isolation of a loved one with dementia related to COVID-19 both impacted their perceptions of the care their loved one was receiving and affected the caregivers’ mental health beyond what they would experience in a normal context. This provides important information about the potential impact of isolation due to COVID-19 for individuals with ADRD and their caregivers.

Our results indicate an inverse relationship between SMD app use and poor mental health. Specifically, *fewer* apps (<2) utilized is associated with a greater number of mental health issues. This is a very preliminary finding and so its definitive indications cannot be determined. However, we will posit and discuss a suspected rationale. Smart personal device apps have been shown to be useful among dementia caregivers (Yousaf et al., 2019; Faieta et al., 2021) and many apps offer features that can be useful for communicating or caring at a distance. Therefore, it is possible that using two or more apps is reflective of a caregiver’s technological ability and comfort level with technology. Caregivers who are more comfortable with smart personal technology can utilize numerous distance communication methods–messaging, video chat, social media, email, etc. Many of the qualitative comments indicated that inability to monitor the care of a loved one with dementia was a source of stress and worry. Caregivers who reached out to their loved ones or to care-facility staff *via* SMDs may have experienced a greater level of control or empowerment when using technology mediated communication, rather than continuing in unremitting isolation from their care recipient. SMD communication apps can be used between caregiver and care recipient and also between caregiver and medical staff. Improving communication through app use might reduce feelings of anxiety and helplessness experienced by caregivers by providing alternative methods of maintaining involvement in provision of care.

The impact of SMD apps as methods of distance communication or caregiver supports is contingent on a number of internal and external factors that affect the human-technology-interface. While information and communication technologies have been found to yield positive outcomes, the ability of communication technologies to mitigate social isolation and support connectedness have not yet been found to *consistently* persist beyond 6 months and that they are not necessarily suitable solutions for every older adult (Chen and Schulz, 2016). Technology evaluation criteria include accessibility factors–for example, is the SMD app visually accessible, does the interface require a certain level of hand dexterity to use? Chen and Schulz (2016) noted that the suitability of information and communication technologies may be impacted by things such as “interest in ICT, motivations for ICT use, cognitive capability, sufficient eyesight, and basic physical ability to use the equipment (e.g., figure or hand movement, skills of using the touch pad).” Other criteria include external and context related factors such affordability, dependability, and learnability (Batavia and Hammer, 1990).

The post-pandemic context is anticipated propel further research into the integration of SMD apps into healthcare and health maintenance. This must be done in light of various usability and accessibility factors as they fluctuate across user groups. Specifically, technology design, research, and implementation should take the unique needs and experiences of older adults into account as well as considerations such as loneliness, economic and environmental factors, and technological ability level (Conroy et al., 2020). Older adults, caregivers, and individuals with dementia in isolated situations may lack informed technology recommendation and adequate support in technology use. Lack of guidance and support can create situations in which apps seem to be hopeful solutions to overcoming communication barriers, but instead prove to be ineffective and lead to further disappointment. Conroy et al. (2020) suggests that technical and scheduling support be offered at the family, care provider, and organizational level to enable older adults to utilize technologies to mitigate the experience of loneliness.

There are numerous factors that can contribute to matches between technology and user, such as the design of the technology, ability to assess the technology, lack of education on technology use–each factor likely impacted by the lack of older adult involvement in the research and design of pervasive technologies such as SMD apps. Sufficient inclusion of older adults as a “target consumer” of general consumer-level, SMDs has yet to be enacted. Mannheim et al. (2019) describes a “gray digital divide” barring older adults from digital technology research and design. Increased education and awareness regarding the equitable inclusion of broad age demographics into the research and design process is needed in order to bridge this divide. This is not to say that research into technology for older adults will not be without challenge–to include methodological barriers like high dropout rates and semantical challenges such as use of a consistent definition of social isolation throughout supporting literature (Chen and Schulz, 2016). Continued interdisciplinary approaches and innovative solutions are needed to overcome these and other barriers to ensure that older adults with ADRD are supported in the pandemic, post-pandemic, and recovery environments.

In sum, there is great potential for SMD apps to influence the quality of life for those impacted by ADRD by facilitating distance communication and care options. SMD apps that can be used by the ADRD or ADRD caregiver populations should be designed with the unique needs and experiences of these groups in mind. The impact of technology on health and quality of life can be influenced by implementation methods, and players–in this case referring to the individual with ADRD, the informal caregiver(s), and staff members at the respective care-facility of residence. Future research should utilize participatory design methods to both (1) develop and identify useful SMD apps to combat social isolation between a caregiver and ADRD care recipient in care-facility settings, and (2) develop end-user sensitive implementation strategies to ensure SMD apps are impactful.

### Limitations

The survey used was designed for the purposes of the present study, therefore it is not a validated instrument. Importantly, this study is exploratory in nature and was used to gather as much insight as possible in a critical window, the period of heightened social isolation during the COVID-19 outbreak in 2020. Therefore, the content and language used in the survey prioritized low respondent burden and general applicability (questions would be applicable to as many caregivers as possible). The use of a self-report survey can be viewed as a limitation due to risk of response bias. However, the perceptions of the respondents represent their reality and are likely the driving factors of behavior. Therefore, these perceptions are what we are most interested in finding. In addition, the items in this survey cannot be viewed as holistically reflecting all potential app uses nor all mental health outcome possibilities. Therefore, the findings in this study should be considered against existing literature and augmented with future studies. Finally, the present survey offers preliminary insight into the experiences and perspectives of a limited sample of caregivers. The web-based format of the survey may introduce selection bias toward participants who are more familiar and comfortable with technology. However, due to COVID-19 safety considerations at the time of data collection this was considered the most appropriate method of collection. The anonymity of the survey limited our ability to determine the generalizability of our results. This sample (*N* = 82) cannot be considered generalizable to the general ADRD caregiver population. Future research is needed to investigate (1) the generalizability of our findings, and (2) the experience and perspectives of caregivers that represent specific racial, ethnic, and cultural, and age groups.

## Conclusion

The present study provides insight into the experiences of caregivers of people with ADRD during period of widespread social isolation. The majority of our sample of caregivers reported that the individual they care for was socially isolated due to institutionalization or hospitalization during COVID-19, and over 87% of caregivers experienced negative mental health outcomes beyond what would have been experienced in typical contexts (e.g., situations unaffected by COVID-19 isolation). The majority of caregivers in our sample perceived the need for SMD app use in their situations. Complementing earlier findings, the present study found that absence or more limited SMD app use was associated with poorer mental health outcomes. Future studies should further investigate the extent and generalizability of app impact in social isolation conditions within the dementia community. Additionally, future research should assess the impact of app evaluation to improve the fit between app and user, thereby potentially improving the usability and usefulness of apps to address distance caregiving and communication.

## Data Availability Statement

The datasets presented in this article are not readily available, because, requests to access original data will be evaluated on an individual basis. Requests to access the datasets should be directed to corresponding author KB, krista.best@fmed.ulaval.ca.

## Ethics Statement

The studies involving human participants were reviewed and approved by Centre Intégré Universitaire de Santé et de Services Sociaux de la Capitale-National Ethics Board (Ethics Approval No. CER 2020-1984). The patients/participants provided their informed consent to participate in this study.

## Author Contributions

All authors contributed to this research through conceptualization, data collection or analysis, and to the development of this manuscript.

## Conflict of Interest

The authors declare that the research was conducted in the absence of any commercial or financial relationships that could be construed as a potential conflict of interest.

## Publisher’s Note

All claims expressed in this article are solely those of the authors and do not necessarily represent those of their affiliated organizations, or those of the publisher, the editors and the reviewers. Any product that may be evaluated in this article, or claim that may be made by its manufacturer, is not guaranteed or endorsed by the publisher.
