# Supplementary material for: Can Technology Abate the Experience of Social Isolation for Those Affected by Dementia?
Source: Front Aging Neurosci. 2022 Feb 22;13:779031. doi: 10.3389/fnagi.2021.779031 (PMC8904898; doi:10.3389/fnagi.2021.779031)
Supplement: Supplementary file 1 [file Table_1.docx]

Supplementary Table 1. Disease State of Individual with Dementia

|  | **Frequency** | **Percent** | **Cumulative Percent** |
| --- | --- | --- | --- |
| **Cognitive deficits from head injury** | 1 | 1.19 | 1.19 |
| **Corticobasal degeneration** | 1 | 1.19 | 2.38 |
| **Huntington’s disease** | 1 | 1.19 | 3.57 |
| **Late onset dementia** | 1 | 1.19 | 4.76 |
| **Late stage dementia** | 1 | 1.19 | 5.95 |
| **Lewy Bodies** | 1 | 1.19 | 7.14 |
| **Mild Alzheimer’s** | 9 | 10.71 | 17.86 |
| **Mixed** | 3 | 3.57 | 21.43 |
| **Mod Alzheimer’s** | 29 | 34.52 | 55.95 |
| **Other** | 4 | 4.76 | 60.71 |
| **Severe Alzheimer’s** | 31 | 36.90 | 97.62 |
| **Vascular Dementia** | 1 | 1.19 | 98.81 |
| **Parkinson’s related** | 1 | 1.19 | 100.00 |
