# Supplementary material for: Can Technology Abate the Experience of Social Isolation for Those Affected by Dementia?
Source: Front Aging Neurosci. 2022 Feb 22;13:779031. doi: 10.3389/fnagi.2021.779031 (PMC8904898; doi:10.3389/fnagi.2021.779031)
Supplement: Supplementary file 2 [file Image_1.pdf]

Using technology and the needs of caregivers of people with Alzheimer's disease during the COVID-19 pandemic

This survey is part of a larger study to further develop a health application review tool (app) to support clinicians to select them to help their Alzheimer's disease (AD) patients and caregivers improve their health and day-to-day functioning. The overall objective of this study is to conduct a methodological evaluation of the Health Applications Review Tool (HART) to develop a matching tool that is both clinically feasible and specifically focused on the needs and experiences of the AD population.

Given the unprecedented impact that COVID-19 has had on health care delivery, it is important to understand how technology can be used to help and caregiver people with AD (while maintaining social distance where possible). Through this survey, we would like to study the impact that COVID-19 has had on the relationship between caregivers of people with AD (or other dementias), perceptions and use of mobile device (i.e. smartphones and tablets).

This survey will take about 5-10 minutes to complete and will include issues related to social distancing/isolation and how mobile device applications have been or could be useful in maintaining health and quality of life in these situations. Your survey responses will be used to help us better understand how to support people with AD or other dementias and their caregivers in times of social isolation and widespread epidemics.

This project has been approved by the Research Ethics Committee for Rehabilitation and Social Integration of CIUSSS of the Capitale-Nationale: Approval No. CER 2020-1984. If you need this survey in a more accessible format or if you have any questions, please contact lead researcher Dr. Julie Faieta at Julie.faieta.1@ulaval.ca. If you have any questions regarding the research project or if you wish to withdraw, you may contact Ms. Krista Best, Researcher and Project Leader at 418-529-9141 ext. 6041 or by email at krista.best@fmed.ulaval.ca. Thank you for your time. [To see the survey in French, please click here- https://www.limesurvey.cifss.ulaval.ca/index.php/323279/lang-fr]

There are 11 questions in this survey

Consent

Privacy

The data collected in this study is completely confidential and will not identify you. The results of the research, which can be disseminated in the form of communications or publications, will not identify participants.

PARTICIPATION

This project has been approved by the Research Ethics Committee for Rehabilitation and Social Integration of CIUSSS of the Capitale-Nationale: Approval No. CER 2020-1984. Your participation in this study is voluntary. You are completely free to participate or not, refuse to answer certain questions or withdraw at any time without prejudice and without having to provide explanations. Whether or not you participate in this study will have no impact on your professional activities. By following the "Continue the survey" link and participating in this survey, you indicate your informed consent to this study.

If you need this survey in a more accessible format or if you have any questions, please contact lead researcher Dr. Julie Faieta at Julie.faieta.1@ulaval.ca. If you have any questions regarding the research project or if you wish to withdraw, you may contact Ms. Krista Best, Researcher and Project Leader at 418-529-9141 ext. 6041 or by email at krista.best@fmed.ulaval.ca. Thank you for your time.

[To see the survey in French, please click here- https://www.limesurvey.cifss.ulaval.ca/index.php/323279/lang-fr]

Please choose **only one** of the following:

- Continue to the survey
- I do not wish to participate in this survey

What is your relationship with someone with Alzheimer's disease or other dementia:

Only answer this question if the following conditions are met:
Answer was 'Continue to the survey ' at question '1 [Consent]' (Privacy The data collected in this study is completely confidential and will not identify you. The results of the research, which can be disseminated in the form of communications or publications, will not identify participants. PARTICIPATION This project has been approved by the Research Ethics Committee for Rehabilitation and Social Integration of CIUSSS of the Capitale-Nationale: Approval No. CER 2020-1984. Your participation in this study is voluntary. You are completely free to participate or not, refuse to answer certain questions or withdraw at any time without prejudice and without having to provide explanations. Whether or not you participate in this study will have no impact on your professional activities. By following the "Continue the survey" link and participating in this survey, you indicate your informed consent to this study. If you need this survey in a more accessible format or if you have any questions, please contact lead researcher Dr. Julie Faieta at Julie.faieta.1@ulaval.ca. If you have any questions regarding the research project or if you wish to withdraw, you may contact Ms. Krista Best, Researcher and Project Leader at 418-529-9141 ext. 6041 or by email at krista.best@fmed.ulaval.ca. Thank you for your time. [To see the survey in French, please click here- https://www.limesurvey.cifss.ulaval.ca/index.php/323279/lang-fr])

Comment only when you choose an answer.

Please choose all that apply and provide a comment:

- [ ] Spouse
- [ ] Adult child
- [ ] Adult grandchild
- [ ] Others, please specify:

Do you care for the person with Alzheimer's disease (AD) or other dementia?

Only answer this question if the following conditions are met:
Answer was 'Continue to the survey ' at question '1 [Consent]' (Privacy The data collected in this study is completely confidential and will not identify you. The results of the research, which can be disseminated in the form of communications or publications, will not identify participants. PARTICIPATION This project has been approved by the Research Ethics Committee for Rehabilitation and Social Integration of CIUSSS of the Capitale-Nationale: Approval No. CER 2020-1984. Your participation in this study is voluntary. You are completely free to participate or not, refuse to answer certain questions or withdraw at any time without prejudice and without having to provide explanations. Whether or not you participate in this study will have no impact on your professional activities. By following the "Continue the survey" link and participating in this survey, you indicate your informed consent to this study. If you need this survey in a more accessible format or if you have any questions, please contact lead researcher Dr. Julie Faieta at Julie.faieta.1@ulaval.ca. If you have any questions regarding the research project or if you wish to withdraw, you may contact Ms. Krista Best, Researcher and Project Leader at 418-529-9141 ext. 6041 or by email at krista.best@fmed.ulaval.ca. Thank you for your time. [To see the survey in French, please click here- https://www.limesurvey.cifss.ulaval.ca/index.php/323279/lang-fr])

Please choose **only one** of the following:

- Yes
- No

Care includes assisting with daily activities, managing medication, travelling in the community, navigating the health care system or leaving a companion for at least 30 minutes, at least 1 day per week (on average).

How would you describe the severity of the illness of the person for whom you are caring?

Only answer this question if the following conditions are met:
Answer was 'Continue to the survey ' at question '1 [Consent]' (Privacy The data collected in this study is completely confidential and will not identify you. The results of the research, which can be disseminated in the form of communications or publications, will not identify participants. PARTICIPATION This project has been approved by the Research Ethics Committee for Rehabilitation and Social Integration of CIUSSS of the Capitale-Nationale: Approval No. CER 2020-1984. Your participation in this study is voluntary. You are completely free to participate or not, refuse to answer certain questions or withdraw at any time without prejudice and without having to provide explanations. Whether or not you participate in this study will have no impact on your professional activities. By following the "Continue the survey" link and participating in this survey, you indicate your informed consent to this study. If you need this survey in a more accessible format or if you have any questions, please contact lead researcher Dr. Julie Faieta at Julie.faieta.1@ulaval.ca. If you have any questions regarding the research project or if you wish to withdraw, you may contact Ms. Krista Best, Researcher and Project Leader at 418-529-9141 ext. 6041 or by email at krista.best@fmed.ulaval.ca. Thank you for your time. [To see the survey in French, please click here- https://www.limesurvey.cifss.ulaval.ca/index.php/323279/lang-fr])

Comment only when you choose an answer.

Please choose all that apply and provide a comment:

- [ ] Mild Alzheimer's disease or other dementia
- [ ] Moderate Alzheimer's disease or other dementia
- [ ] Severe Alzheimer's disease or other dementia
- [ ] If other dementias, please specify the type of dementia

Was this person isolated from you in a hospital or care facility because of the COVID-19 pandemic?

Only answer this question if the following conditions are met:
Answer was 'Continue to the survey ' at question '1 [Consent]' (Privacy The data collected in this study is completely confidential and will not identify you. The results of the research, which can be disseminated in the form of communications or publications, will not identify participants. PARTICIPATION This project has been approved by the Research Ethics Committee for Rehabilitation and Social Integration of CIUSSS of the Capitale-Nationale: Approval No. CER 2020-1984. Your participation in this study is voluntary. You are completely free to participate or not, refuse to answer certain questions or withdraw at any time without prejudice and without having to provide explanations. Whether or not you participate in this study will have no impact on your professional activities. By following the "Continue the survey" link and participating in this survey, you indicate your informed consent to this study. If you need this survey in a more accessible format or if you have any questions, please contact lead researcher Dr. Julie Faieta at Julie.faieta.1@ulaval.ca. If you have any questions regarding the research project or if you wish to withdraw, you may contact Ms. Krista Best, Researcher and Project Leader at 418-529-9141 ext. 6041 or by email at krista.best@fmed.ulaval.ca. Thank you for your time. [To see the survey in French, please click here- https://www.limesurvey.cifss.ulaval.ca/index.php/323279/lang-fr])

Please choose **only one** of the following:

- Yes
- No

If your answer to number 4 was yes, please respond to the following based on your experiences when you were isolated from that person. If your answer was no, please answer the following question based on what you think your experiences would be if you were socially isolated from that person.

Have you had any concerns about the care that medical and/or care personnel are able to provide to the person with AD or any other dementia during the COVID-19 isolation period?

Only answer this question if the following conditions are met:
Answer was 'Continue to the survey ' at question '1 [Consent]' (Privacy The data collected in this study is completely confidential and will not identify you. The results of the research, which can be disseminated in the form of communications or publications, will not identify participants. PARTICIPATION This project has been approved by the Research Ethics Committee for Rehabilitation and Social Integration of CIUSSS of the Capitale-Nationale: Approval No. CER 2020-1984. Your participation in this study is voluntary. You are completely free to participate or not, refuse to answer certain questions or withdraw at any time without prejudice and without having to provide explanations. Whether or not you participate in this study will have no impact on your professional activities. By following the "Continue the survey" link and participating in this survey, you indicate your informed consent to this study. If you need this survey in a more accessible format or if you have any questions, please contact lead researcher Dr. Julie Faieta at Julie.faieta.1@ulaval.ca. If you have any questions regarding the research project or if you wish to withdraw, you may contact Ms. Krista Best, Researcher and Project Leader at 418-529-9141 ext. 6041 or by email at krista.best@fmed.ulaval.ca. Thank you for your time. [To see the survey in French, please click here- https://www.limesurvey.cifss.ulaval.ca/index.php/323279/lang-fr])

Please choose **only one** of the following:

- Yes
- No

For example, have you had concerns about the person with AD or other dementia who can communicate their needs without you being physically present to help them?

Has the isolation of a person with AD or other dementia (which you are caring for) related to COVID-19 increased your need for mobile device (i.e. smartphones and tablets) use?

Only answer this question if the following conditions are met:
Answer was 'Continue to the survey ' at question '1 [Consent]' (Privacy The data collected in this study is completely confidential and will not identify you. The results of the research, which can be disseminated in the form of communications or publications, will not identify participants. PARTICIPATION This project has been approved by the Research Ethics Committee for Rehabilitation and Social Integration of CIUSSS of the Capitale-Nationale: Approval No. CER 2020-1984. Your participation in this study is voluntary. You are completely free to participate or not, refuse to answer certain questions or withdraw at any time without prejudice and without having to provide explanations. Whether or not you participate in this study will have no impact on your professional activities. By following the "Continue the survey" link and participating in this survey, you indicate your informed consent to this study. If you need this survey in a more accessible format or if you have any questions, please contact lead researcher Dr. Julie Faieta at Julie.faieta.1@ulaval.ca. If you have any questions regarding the research project or if you wish to withdraw, you may contact Ms. Krista Best, Researcher and Project Leader at 418-529-9141 ext. 6041 or by email at krista.best@fmed.ulaval.ca. Thank you for your time. [To see the survey in French, please click here- https://www.limesurvey.cifss.ulaval.ca/index.php/323279/lang-fr])

Please choose **only one** of the following:

- Yes
- No

For example, have you used mobile device (i.e. smartphones and tablets) to communicate with the person with AD or other dementia or with their clinicians? Have you used mobile device (i.e. smartphones and tablets) to monitor their care remotely?

If so at number 6, what applications have you used in relation to the person with AD or other dementia?

Only answer this question if the following conditions are met:
Answer was 'Continue to the survey ' at question '1 [Consent]' (Privacy The data collected in this study is completely confidential and will not identify you. The results of the research, which can be disseminated in the form of communications or publications, will not identify participants. PARTICIPATION This project has been approved by the Research Ethics Committee for Rehabilitation and Social Integration of CIUSSS of the Capitale-Nationale: Approval No. CER 2020-1984. Your participation in this study is voluntary. You are completely free to participate or not, refuse to answer certain questions or withdraw at any time without prejudice and without having to provide explanations. Whether or not you participate in this study will have no impact on your professional activities. By following the "Continue the survey" link and participating in this survey, you indicate your informed consent to this study. If you need this survey in a more accessible format or if you have any questions, please contact lead researcher Dr. Julie Faieta at Julie.faieta.1@ulaval.ca. If you have any questions regarding the research project or if you wish to withdraw, you may contact Ms. Krista Best, Researcher and Project Leader at 418-529-9141 ext. 6041 or by email at krista.best@fmed.ulaval.ca. Thank you for your time. [To see the survey in French, please click here- https://www.limesurvey.cifss.ulaval.ca/index.php/323279/lang-fr])

Comment only when you choose an answer.
Please select at most 6 answers

Please choose all that apply and provide a comment:

- [ ] Videoconferencing application
- [ ] Messaging app
- [ ] Apps to put you in touch with a health care provider (for example, my file, other example (in case they don't know this one))
- [ ] Internet browser applications (e.g., Safari, Chrome, Internet Explorer)
- [ ] Others, please specify:
- [ ] None, I only used voice calls

Has the isolation of a person with AD or other dementia (of which you are involved) related to COVID-19 affect your mental health beyond the usual health and well-being concerns of that person?

Only answer this question if the following conditions are met:
Answer was 'Continue to the survey ' at question '1 [Consent]' (Privacy The data collected in this study is completely confidential and will not identify you. The results of the research, which can be disseminated in the form of communications or publications, will not identify participants. PARTICIPATION This project has been approved by the Research Ethics Committee for Rehabilitation and Social Integration of CIUSSS of the Capitale-Nationale: Approval No. CER 2020-1984. Your participation in this study is voluntary. You are completely free to participate or not, refuse to answer certain questions or withdraw at any time without prejudice and without having to provide explanations. Whether or not you participate in this study will have no impact on your professional activities. By following the "Continue the survey" link and participating in this survey, you indicate your informed consent to this study. If you need this survey in a more accessible format or if you have any questions, please contact lead researcher Dr. Julie Faieta at Julie.faieta.1@ulaval.ca. If you have any questions regarding the research project or if you wish to withdraw, you may contact Ms. Krista Best, Researcher and Project Leader at 418-529-9141 ext. 6041 or by email at krista.best@fmed.ulaval.ca. Thank you for your time. [To see the survey in French, please click here- https://www.limesurvey.cifss.ulaval.ca/index.php/323279/lang-fr])

Please choose **only one** of the following:

- Yes
- No

If so at number 8, how has your mental health been affected?

Only answer this question if the following conditions are met:
Answer was 'Continue to the survey ' at question '1 [Consent]' (Privacy The data collected in this study is completely confidential and will not identify you. The results of the research, which can be disseminated in the form of communications or publications, will not identify participants. PARTICIPATION This project has been approved by the Research Ethics Committee for Rehabilitation and Social Integration of CIUSSS of the Capitale-Nationale: Approval No. CER 2020-1984. Your participation in this study is voluntary. You are completely free to participate or not, refuse to answer certain questions or withdraw at any time without prejudice and without having to provide explanations. Whether or not you participate in this study will have no impact on your professional activities. By following the "Continue the survey" link and participating in this survey, you indicate your informed consent to this study. If you need this survey in a more accessible format or if you have any questions, please contact lead researcher Dr. Julie Faieta at Julie.faieta.1@ulaval.ca. If you have any questions regarding the research project or if you wish to withdraw, you may contact Ms. Krista Best, Researcher and Project Leader at 418-529-9141 ext. 6041 or by email at krista.best@fmed.ulaval.ca. Thank you for your time. [To see the survey in French, please click here- https://www.limesurvey.cifss.ulaval.ca/index.php/323279/lang-fr])

Comment only when you choose an answer.
Please select at most 7 answers

Please choose all that apply and provide a comment:

- [ ] Increased anxiety
- [ ] Increased stress
- [ ] Increased perceived load
- [ ] Decreased anxiety
- [ ] Reduced stress
- [ ] Decrease in perceived load
- [ ] Others, please specify:

If you answered yes to number 8, has the use of mobile device (i.e. smartphones and tablets) apps to communicate with your loved one with AD or some other form of dementia have an impact on your mental health?

Only answer this question if the following conditions are met:
Answer was 'Continue to the survey ' at question '1 [Consent]' (Privacy The data collected in this study is completely confidential and will not identify you. The results of the research, which can be disseminated in the form of communications or publications, will not identify participants. PARTICIPATION This project has been approved by the Research Ethics Committee for Rehabilitation and Social Integration of CIUSSS of the Capitale-Nationale: Approval No. CER 2020-1984. Your participation in this study is voluntary. You are completely free to participate or not, refuse to answer certain questions or withdraw at any time without prejudice and without having to provide explanations. Whether or not you participate in this study will have no impact on your professional activities. By following the "Continue the survey" link and participating in this survey, you indicate your informed consent to this study. If you need this survey in a more accessible format or if you have any questions, please contact lead researcher Dr. Julie Faieta at Julie.faieta.1@ulaval.ca. If you have any questions regarding the research project or if you wish to withdraw, you may contact Ms. Krista Best, Researcher and Project Leader at 418-529-9141 ext. 6041 or by email at krista.best@fmed.ulaval.ca. Thank you for your time. [To see the survey in French, please click here- https://www.limesurvey.cifss.ulaval.ca/index.php/323279/lang-fr])

Comment only when you choose an answer.
Please select at most 6 answers

Please choose all that apply and provide a comment:

- [ ] Yes, it reduced my anxiety (positive impact)
- [ ] Yes, it reduced my stress (positive impact)
- [ ] Yes, this has reduced the load I perceive (positive impact)
- [ ] No, it increased my anxiety (negative impact)
- [ ] No, it had no impact (neutral impact)
- [ ] Not applicable, I do not, or would not, use a mobile device (i.e. smartphones and tablets) in this situation

We thank you for your willingness to participate in this survey and hope that the results will improve the care of people with Alzheimer's disease and other dementias.

09-13-2020 – 09:31

Submit your survey.
Thank you for completing this survey.
