# Supplementary material for: Can Technology Abate the Experience of Social Isolation for Those Affected by Dementia?
Source: Front Aging Neurosci. 2022 Feb 22;13:779031. doi: 10.3389/fnagi.2021.779031 (PMC8904898; doi:10.3389/fnagi.2021.779031)
Supplement: Supplementary file 3 [file Image_2.pdf]

Appendix B

Sample Participant Comments Related to Experience of Isolation from Care Recipients

- *"Unsure she will be fed properly with being confined"*
- *"Limited time. Promises to call back and don't. Seeing my mom agitated makes me stressed"*
- *"I have lupus, this lock out has caused more stress than I have ever had!! And I'm a retired K-9 officer"*
- *"heartbroken, sleep deprived, depression, separation anxiety and constant concern"*
- *"Terrible anxiety that I'll never see her again"*
- *"Anxious that she dies alone"*
- *"Can't sleep at night worried about her care"*
- *"increased stress due to my parents' separation"*
- *"I couldn't see the facial expressions to decipher his feelings. I had to evaluate the tone of his voice."*
- "*With the camera, I saw him wither away. Not [because] slept" [Avec la caméra, je l'ai vu déperrir. pas bcp dormi]*
- *"It can be all consuming, worrying about her well-being and feeling completely helpless"*
- *"more work for me since COVID. during isolation, I took many calls. Since access, I coordinate COVID testing and visits for my Dad."*
